# “Net cage” technique in the treatment of inferior pole patella fracture

**DOI:** 10.3389/fsurg.2026.1648538

**Published:** 2026-03-11

**Authors:** Hongfei Qi, Zhong Li, Hua Lin, Shuai Ji, Chengcheng Zhang, Bo Wu, Bing Du, Kun Zhang, Ming Li

**Affiliations:** Honghui Hospital, Xi'an Jiaotong University, Xi’an, Shaanxi, China

**Keywords:** avulsion fractures, complications, inferior patellar pole, modified tension-band, radiological

## Abstract

**Objective:**

: Fractures of the inferior pole of the patella can cause disorders of the knee joint extensor mechanism. The fracture fragments are usually small and comminuted, presenting certain difficulties in fixation. The purpose of this study is to observe the clinical effect of the “Net cage” technique in the treatment of inferior pole patella fractures.

**Methods:**

This is a retrospective study that included 16 cases of inferior patella fractures (AO/OTA 34-A1) who underwent the “Net cage” technique from March 2017 to June 2020. Their medical records and follow-up results were collected, and the measured indicators included surgical complications related to the fixation method, knee joint function, the number of fluoroscopies, fracture healing, and the incidence of soft tissue stimulation.

**Results:**

All patients achieved smooth fracture healing without complications such as internal fixation failure or implant fracture. The average number of intraoperative fluoroscopies was 5.56 ± 1.82 times (range: 4–10 times); the average fracture healing time was 10.5 ± 1.96 weeks (range: 8–14 weeks). No patients reported internal fixation-related soft tissue irritation. At the last follow-up, the knee joint function evaluation showed that the average range of motion (ROM) was 133.75 ± 5.89° (range: 120°∼140°); the average Bostman score was 27.94 ± 1.83 points (range: 24–30 points). Due to the small sample size of this study, only descriptive statistical analysis was performed on the data.

**Conclusion:**

The “Net cage” technique for the treatment of inferior pole patella fractures shows promising short- to mid-term results, with the potential advantages of reducing fluoroscopy times, providing stable fixation, facilitating early functional exercise, and achieving favorable postoperative knee joint function. However, given the limitations of the study design, the clinical application and advantages of this technique require further verification by large-sample, multi-center randomized controlled studies.

## Introduction

Fractures of the inferior patellar pole account for approximately 20% of all surgically managed patellar fractures (1), and they typically result from direct trauma to the flexed knee joint (2). Similar to other types of patellar fractures, the primary goal of surgical intervention for inferior pole fractures is to reconstruct the extensor mechanism while restoring the stability of the articular surface (3); However, a key distinction lies in the fact that fracture fragments of the inferior patellar pole are often small and comminuted, which poses significant challenges to maintaining effective reduction and stable fixation (4–6). To address the clinical challenge of achieving stable fixation for inferior patellar pole fractures, a variety of novel fixation strategies have been developed and validated in clinical practice, including suture fixation, plate fixation, and plate-integrated tension-band fixation (7–9). Additionally, partial patellectomy combined with patellar ligament repair and anchor suture fixation has been adopted for the management of comminuted distal patellar pole fractures (9, 10), However, the clinical efficacy and generalizability of this latter approach remain insufficiently validated, and it has not yet gained widespread acceptance within the orthopedic community.

However, in the context of comminuted fractures of the inferior patellar pole, traditional tension-band fixation techniques are associated with a relatively high risk of fracture fragment displacement and internal fixation failure (6). To address these clinical challenges, we have developed an innovative “Net cage” fixation technique. Conceptually, this technique draws inspiration from the net cage structures used in zoos to confine large animals such as lions. Specifically, we utilize titanium cables and bone pins to construct a “Net cage” structure that encapsulates and stabilizes the fracture fragments of the inferior patellar pole, thereby enabling the reconstruction of the knee extensor mechanism. The incorporation of two sets of tension bands minimizes gaps within the “Net cage” structure, effectively preventing the escape of small fracture fragments. This dual-tension-band design ensures reliable stabilization of the inferior patellar pole fracture and has yielded favorable clinical outcomes in our clinical practice. The detailed methodology and relevant clinical findings of this technique are presented in this study.

## Patients and methods

### Study design and patient enrollment

**Figure 1:**
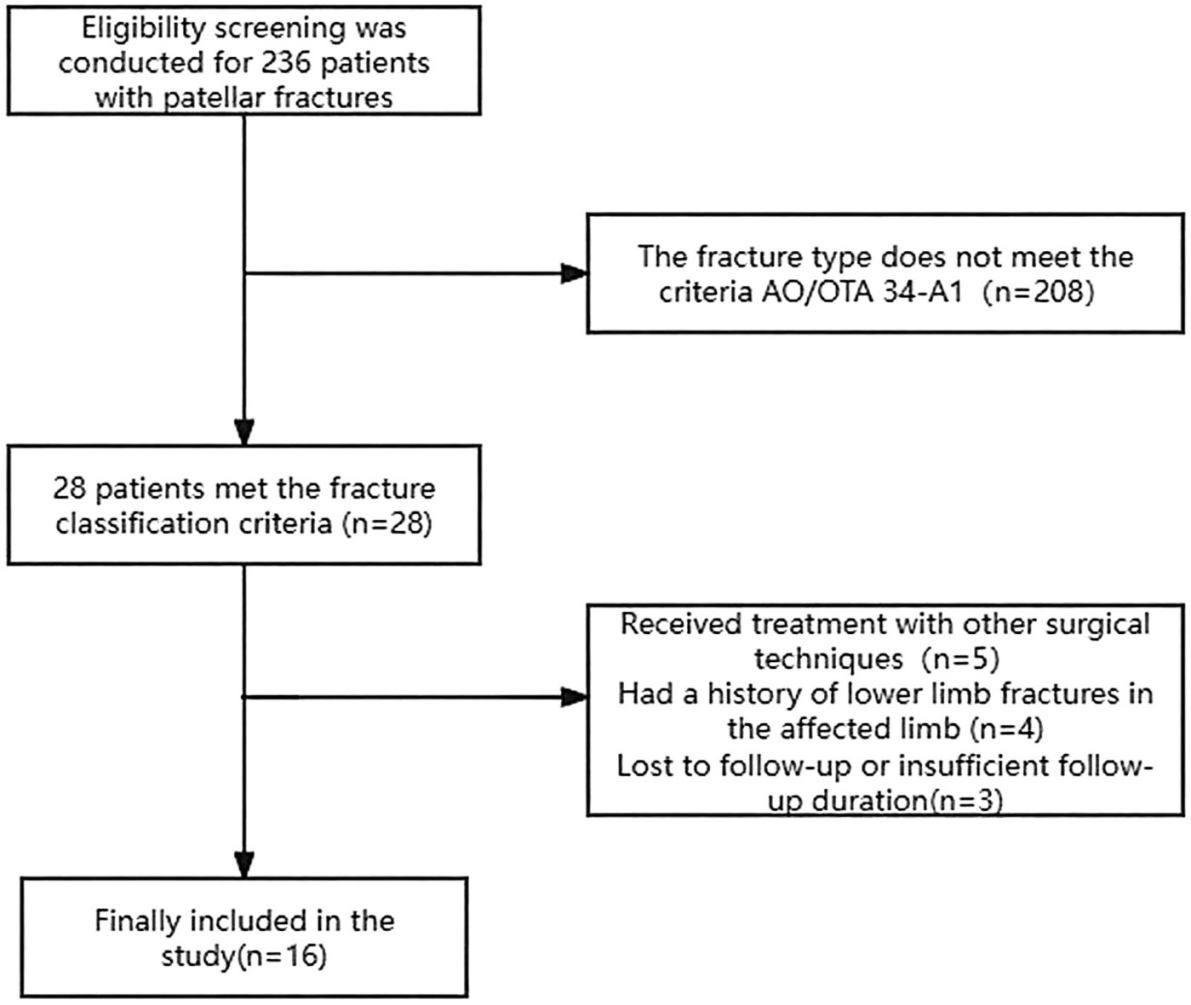
Investigator inclusion flow chart.

This study was designed as a retrospective analysis. From March 2017 to June 2020, a total of 236 patients with patellar fractures were admitted to Hong Hui Hospital of Xi'an Jiaotong University and underwent surgical treatment. Among these patients, 28 were diagnosed with inferior patellar pole fractures classified as AO/OTA 34-A1. Patients were excluded based on the following criteria: (1) undergoing partial patellectomy or alternative surgical procedures (*n* = 5); (2) concurrent femoral or tibial fractures that might interfere with postoperative knee joint function assessment (*n* = 4); (3) incomplete follow-up data (*n* = 3). Finally, 16 patients who received the “Net cage” fixation technique and completed a minimum of 12 months of follow-up were included in the final analysis. The patient enrollment flowchart is presented in Figure 1.

All patients underwent comprehensive preoperative evaluations, including standard anteroposterior and lateral knee x-rays, computed tomography (CT) scanning, and three-dimensional reconstruction. These imaging modalities were used to assess the severity of fracture comminution and confirm fracture classification. All surgical procedures were performed by a single orthopedic surgeon with extensive experience in trauma management to ensure consistency in surgical technique. Postoperative functional assessments were conducted by independent professional rehabilitation physicians who were not involved in the surgical treatment or study design, thus eliminating potential assessment bias.

This study was conducted in strict adherence to the ethical principles outlined in the Declaration of Helsinki. The study protocol was reviewed and approved by the Ethics Committee of Hong Hui Hospital Affiliated to Xi'an Jiaotong University (Approval No.: 20170222; Xi'an, Shaanxi, China). Written informed consent was obtained from all patients prior to their participation in the study, including consent for the use of their clinical data and follow-up information for research purposes.

### Surgical technique

The patient was placed in a supine position under general anesthesia, antibiotics were used prophylactically, and then a tourniquet was placed on the base of the patient's thigh. The pressure of the tourniquet was 300mmHg. Surgical incisions are selected from the anterior median longitudinal incision of the knee joint, and the skin, subcutaneous tissue, and superficial fascia are cut layer by layer to remove blood clots between the fractures, avoiding the peeling of the prepatellar fascia and periosteum to prevent the separation of comminuted fractures.

**Figure 2:**
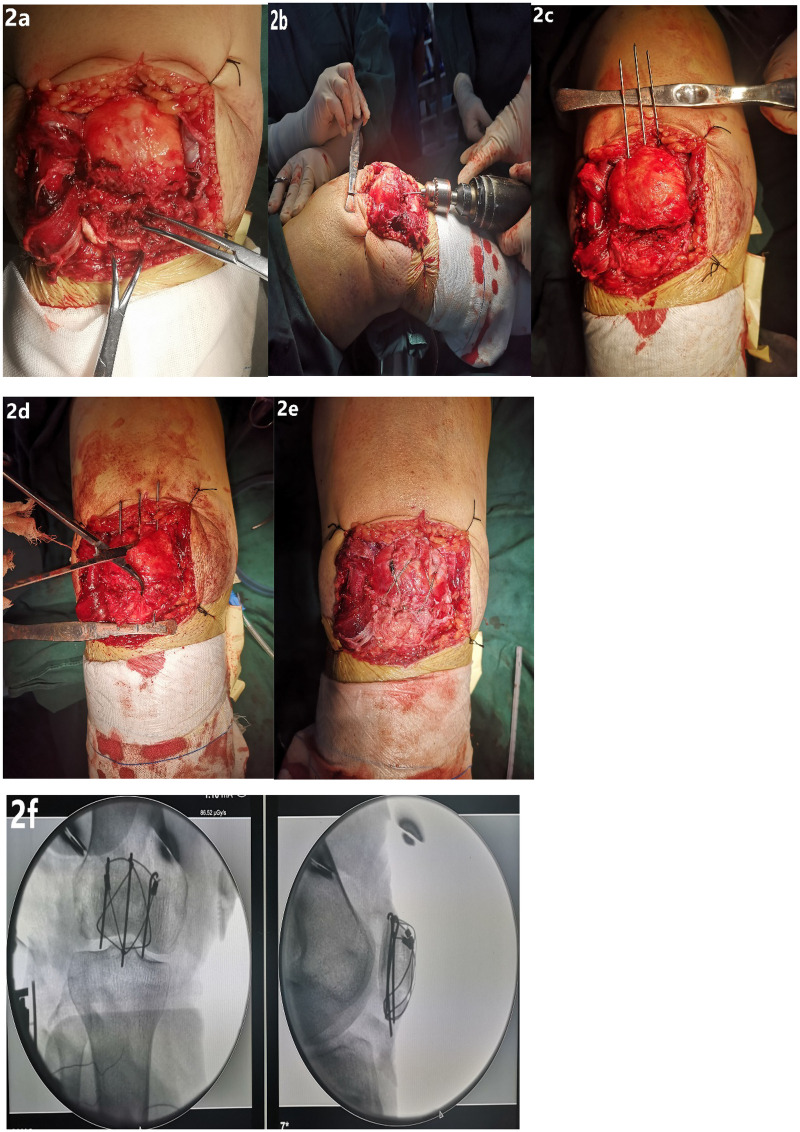
**(a–c)** Three 1.5 K-wires were driven from the bottom to the top of the fracture end of the lower pole of the patella; **(d)** then pierce the K-wire from top to bottom, and make three K-wires support and fix the bone needle block at the lower pole of patella; **(e)** tighten the steel cable to form “net cage” structure to fix the fracture of the lower pole of the patella; **(f)** C-arm fluoroscopy showed satisfactory reduction and firm internal fixation of the fracture.

After the fractured end is revealed, three 1.5 mm K-wires were driven parallel from the subchondral bone area of the fracture section to the upper pole of the patella (Figures 2a–c, during this process, you can directly touch the articular cartilage surface of the patella with your hands to assist in determining the position of the K-wire). Use Bone tenaculum to maintain the reduction of the fracture of the inferior pole of the patella and pass the 3 K-wire retrogradely out of the patella ligament, so that the K-wire is supported under the bone block of the inferior pole in a row of needles (Figure 2d), bend the tail of the K-wire at the upper pole of the patella for 180°, pull down the K-wire, and buckle the hook end of the K-wire at the upper pole of the patella into the quadriceps femoris tendon, and then use two steel cables or steel wires to cross in pairs to fix the fracture in a “net cage” technique (Figure 2e). Re-check the reduction of the fracture through the C-arm fluoroscopy (Figure 2f) and flex the knee to check the stability of the inferior pole fracture. After confirming satisfaction, the drainage tube is left in the wound, and the wound is closed layer by layer. Two details in the surgical procedure deserve attention. First, the second cable should be placed under the first one using a cable passer. This way, after the cables are tightened, the two tension bands can work synergistically—even if one tension band fails, the other can provide protection to avoid complete failure (see Supplementary Materials). Second, when fixing, the cable at the inferior pole should pass through part of the patellar tendon, which can reduce the risk of the cable dislodging from the hook at the inferior pole of the patella during knee flexion.

After the fracture has healed, for patients who have a strong request to remove the internal fixation, we only need to cut the upper part to expose the wire cable tightening device or wire knot and remove the K-wire, so we need to pay attention to the placement of the wire cable tightening device or wire knot on the upper part of the patella.

### Rehabilitation protocol

The postoperative rehabilitation of patellar fracture is implemented in phases: During Weeks 1–2, a brace locked at 30° is worn, with passive range of motion (ROM) ≥45° and active ROM ≥30°; quadriceps isometric contraction training (3 sets × 10 repetitions, 5-s hold) is performed, along with partial weight-bearing (20%–30% of body weight). In Weeks 3–4, the brace is adjusted to an unlocked 60° position, with passive and active ROM reaching ≥90° and ≥60° respectively; isotonic contraction training (3 sets × 12 repetitions, 0.5 kg load) is conducted, and weight-bearing is increased to 50%–60% of body weight. For Weeks 5–6, the brace is removed to allow full-range knee movement, with passive ROM ≥120° and active ROM ≥90°; eccentric training (3 sets × 10 repetitions, 1 kg load) is carried out, accompanied by full weight-bearing (100% of body weight). From Weeks 7–12, brace use is discontinued, with passive ROM ≥135° and active ROM ≥120°; functional training such as squats and step-ups is performed, combined with activity-specific full weight-bearing, to gradually restore knee joint function (for details, refer to the Supplementary File).

### Outcome indicators

The primary outcome measures of this study were surgical complications related to the fixation method and postoperative knee joint function. Specifically, the evaluated complications included internal fixation failure, fracture fragment displacement, implant fracture, and implant dislocation; knee joint function was assessed to reflect the recovery of extensor mechanism and daily mobility. The secondary outcome measures comprised the number of intraoperative fluoroscopies (to quantify radiation exposure) and the incidence of internal fixation-related soft tissue irritation (e.g., local pain, swelling, or discomfort caused by implants).

### Follow-up protocol and assessment methods

All patients were scheduled for regular follow-up visits, with the first follow-up at 4 weeks postoperatively and subsequent visits monthly thereafter until fracture healing was confirmed. During each postoperative follow-up, the following assessments were performed:

Radiological evaluation: Standard anteroposterior and lateral knee x-rays were obtained to monitor fracture reduction maintenance, implant position, and fracture healing progress.
Knee joint function assessment: Knee function was evaluated using two validated metrics at the final follow-up:
Range of Motion (ROM): Patients were instructed to lie in the prone position, and a standard goniometer was used to measure the active and passive ROM of the knee joint (recorded as the range from full extension to maximum flexion).

### Bostman score

This scoring system (range: 0–30) was used to assess overall patellofemoral function, including items such as pain, ROM, swelling, stair-climbing ability, and kneeling capacity; higher scores indicate better functional recovery. The Bostman score was selected as the primary functional assessment tool because it is widely used in clinical practice for evaluating patellar fracture outcomes, with clear scoring criteria and good clinical applicability, enabling straightforward assessment of key functional aspects such as pain, mobility, and daily activity ability.

### Fracture healing criteria

Fracture healing was defined based on a combination of clinical and radiological criteria:

Clinical criteria: Absence of pain or tenderness at the fracture site, and the ability to walk independently for at least 50 m without crutches or other assistive devices.

Radiological criteria: Clear evidence of trabecular continuity between the two main fracture fragments on anteroposterior and lateral knee x-rays, with no visible fracture line or signs of delayed union/nonunion.

## Results

### Patient demographics and baseline clinical characteristics

#### Clinical outcomes

A total of 16 patients were enrolled in this study. Their demographic data, injury mechanisms, and fracture-related characteristics are summarized in Table 1. Among the included patients: 11 were male and 5 were female; the age range was 32–57 years, with a mean age of 41.50 ± 7.39 years; the mechanism of injury was falls in 12 patients and traffic accidents in 4 patients; the mean length of the inferior patellar pole fracture fragment was 10.8 ± 2.13 mm (range: 7.5–13.7 mm).

**Table 1:** Patient demographics and clinical outcomes.

Patient	Age (years)	Sex	Average length of inferior pole fracture fragment(mm)	Union time (weeks)	ROM(°)	Bostman score
1	40	Male	7.9	8	0–135	28
2	32	Female	8.8	12	0–140	28
3	38	Female	11.3	12	0–135	28
4	41	Male	12.5	10	0–140	30
5	42	Female	10.9	10	0–120	30
6	33	Male	13.4	12	0–125	27
7	57	Male	13.7	12	0–135	28
8	47	Female	7.5	8	0–140	28
9	50	Male	13.1	14	0–135	27
10	52	Female	11.2	12	0–130	24
11	33	Male	10.5	10	0–140	27
12	34	Male	8.4	10	0–130	30
13	38	Male	9.6	8	0–135	28
14	37	Male	9.8	8	0–135	27
15	44	Male	11.2	10	0–125	27
16	46	Male	12.4	12	0–140	30

### Fracture healing and follow-up duration

All 16 patients achieved uneventful fracture healing without major complications such as internal fixation failure, implant fracture, or implant dislocation. The mean duration of postoperative follow-up was 14 ± 2.31 months (range: 12–18 months), and the mean fracture healing time was 10.5 ± 1.96 weeks (range: 8–14 weeks).

### Postoperative complications

No patient developed severe postoperative complications, including deep surgical site infection, internal fixation failure, or implant-related soft tissue irritation requiring implant removal. One patient presented with wound fat liquefaction postoperatively; this complication was managed by extending the wound dressing change interval, and the wound eventually healed completely without further sequelae.

### Knee joint function at final follow-up

At the final follow-up, knee joint function assessment of all patients showed favorable recovery: the mean ROM of the knee joint was 133.75 ± 5.89° (range: 120°–140°), and the mean Bostman score was 27.94 ± 1.83 (range: 24–30). Among the patients, 14 achieved a knee ROM comparable to that of the contralateral uninjured knee, and 12 patients obtained a Bostman score of ≥27 (indicating excellent or good functional recovery).

## Discussion

Open reduction and internal fixation (ORIF) remains the mainstay of treatment for inferior patellar pole fractures, with the core goals of restoring quadriceps muscle function and preventing long-term osteoarthritis development (11). However, this treatment strategy faces unique challenges: inferior patellar pole fracture fragments are typically small and highly comminuted, making it difficult to achieve effective and stable internal fixation (6, 11). Additionally, prolonged postoperative brace immobilization—often required to compensate for inadequate fixation—can lead to knee joint stiffness and functional impairment, further compromising patient outcomes. To address the unmet need for stable fixation of inferior patellar pole fractures, a variety of fixation techniques have been explored in clinical practice, yet each has inherent limitations. Lazaro et al. reported (12) reported the use of circular ligation and suture fixation for this fracture type; however, this method fails to effectively appose the proximal articular surface of the patella, increasing the risk of fracture fragments migrating into the joint cavity. Zhu et al. (9) demonstrated that miniplate combined with tension-band wiring achieved moderate clinical efficacy in treating inferior patellar pole fractures. While plate fixation provides a novel approach to fracture stabilization (9, 13, 14), the patella's anatomical characteristics (as a sesamoid bone with an irregular surface) make it challenging to achieve optimal plate positioning. Furthermore, the narrow anatomical space between the inferior patellar pole and the patellar ligament increases the risk of soft tissue injury during plate placement, potentially exacerbating postoperative pain and delaying recovery. Assaf et al. (15) described anchor suture fixation, which involves suturing the inferior pole fracture fragment to the patellar ligament to reconstruct knee extensor function. Nevertheless, this technique often lacks sufficient fixation strength, necessitating prolonged postoperative brace immobilization that hinders early functional exercise—a key factor in preserving knee mobility. Other alternative methods, such as basket plate fixation (13) and X-Change acetabular revision mesh (16) show theoretical promise for comminuted fractures but are limited by complex surgical procedures and increased soft tissue trauma, which may not be suitable for all patients.

Partial patellectomy is another therapeutic option for inferior patellar pole fractures, particularly in cases of severely comminuted fractures where ORIF is deemed technically unfeasible (17–19). However, accumulating evidence has highlighted significant drawbacks of this approach. Kaufer (20) demonstrated that partial patellectomy reduces knee extensor mechanism function by approximately 30%—a permanent deficit that impairs patients' ability to perform activities such as climbing stairs or rising from a seated position. Beyond functional loss, resection of the inferior patellar pole disrupts the anatomical integrity of the extensor mechanism: it alters patellar tendon length, leading to caudal patellar migration, and shifts the patellar ligament insertion posteriorly. These changes disrupt the normal biomechanical distribution of the patellofemoral joint (4, 21), increasing the risk of long-term complications such as patellofemoral pain syndrome, chondromalacia, and accelerated osteoarthritis. Given these adverse outcomes, maximal patellar preservation should be prioritized in the surgical management of inferior patellar pole fractures whenever technically possible.

Tension-band wiring fixation is widely regarded as the gold standard for patellar fractures, as it is associated with optimal postoperative knee function recovery (22). Biomechanically, contraction of the quadriceps muscle generates tensile forces that tend to distract the patellar fracture fragments. Tension-band wiring converts these tensile forces into compressive forces at the fracture site, enabling the bone to withstand high tensile loads and thereby promoting fracture healing (2). Several studies have described the use of anterior tension-band wiring through cannulated screws for the internal fixation of displaced inferior patellar pole fractures, reporting favorable clinical outcomes (6). However, this modified tension-band technique still has significant limitations: it requires a sufficiently large fracture fragment to provide stable anchorage for the cannulated screws and tension band, making it ineffective for small, highly comminuted inferior patellar pole fractures—precisely the subset of fractures that pose the greatest clinical challenge. Furthermore, traditional tension-band wiring fixation itself is associated with a range of complications, including Kirschner wire (K-wire) migration, steel wire breakage, and internal fixation cut-through into the patellar bone. These issues often lead to fixation failure, delayed healing, or the need for revision surgery, further compromising patient outcomes. To address the unmet clinical need for stable fixation of small, comminuted inferior patellar pole fractures—and to overcome the limitations of traditional tension-band techniques—we developed an innovative “Net cage” fixation technique. Through years of clinical application, this technique has consistently yielded favorable outcomes in our practice, including reliable fracture stabilization, low complication rates, and good functional recovery. We therefore believe that the “Net cage” technique provides a valuable alternative approach for the clinical management of inferior patellar pole fractures, particularly in cases where traditional methods are technically unfeasible or associated with high failure risk.

The “Net cage” fixation technique we developed is not a mere modification of traditional dual-set tension-band fixation but a comprehensive innovation tailored to the anatomical and biomechanical characteristics of inferior patellar pole fractures. Its core advantages are elaborated as follows: First, the technique is grounded in a clear anatomical understanding of inferior patellar pole fractures: such fractures can be categorized as a distinct type of avulsion fracture that does not involve the patellar articular cartilage surface. Thus, the primary surgical goal is to reconstruct the continuity between the patella and patellar ligament to restore the knee extensor mechanism—an concept analogous to anchor suture fixation (15). The “Net cage” structure, constructed from bone pins and steel cables, effectively secures the inferior pole fracture fragments and patellar ligament to the proximal patellar fracture end, providing robust fixation strength for the fracture (see Figure 2e). This design directly addresses the key challenge of stabilizing small, comminuted fragments. Secondly, during the operation, we can directly touch the articular surface with our hands during the process of inserting the 3 bone spicules from bottom to top. This not only reduces the number of intraoperative fluoroscopy but also ensures more precise insertion of the bone spicules. Drive 3 bone spicules parallel to the articular surface into the subchondral bone area. This area has a stronger bone condition and reduces the risk of disengagement and failure of the tension band due to the withdrawal of the spicules (due to the injury mechanism, fractures of the inferior pole of the patella are often compressed and comminuted in layers, and the bone mass is poor, which potentially reduces the firmness of the fixation). Meanwhile, the three bone spicules are held parallel under the distal fracture fragments, which is similar to the raft nail effect (23) (similar to the tibial plateau fracture plate) to better support the inferior pole fracture fragments. It forms a cross mesh structure with the two tension band steel cables on the upper surface of the fracture fragments. The three-dimensional wrapping is similar to the “net cage” structure, which firmly hoops the inferior pole fracture fragments on the patella body, to restore the continuity of patellar ligament and reconstruction of knee extension device. Finally, we choose two sets of tension bands for fixation for many reasons: (1) The “net cage” structure composed of the two sets of tension band pins and steel cables is smaller, which can prevent fracture fragments from leaking from the “net cage” structure; (2) One set of tension band fixation is prone to cutting between internal fixation and fracture fragments. When the two sets of tension bands are fixed, the contact area between the lower pole fracture block and the internal fixation increases, and the pressure on the contact surface will decrease accordingly, reducing the occurrence of cutting. (3) Even if one set of tension bands is withdrawn or loosened, the other set of tension bands will still play a role to avoid complete failure of internal fixation. This has been confirmed in 34-C patella fractures.

In our cohort, all 16 patients achieved uneventful healing without fixation failure or implant complications. The mean knee ROM (133.75°) and Bostman score (27.94 points) at follow-up were favorable, with 14 patients recovering ROM comparable to the contralateral limb. These results align with or exceed those of other techniques (9, 13), suggesting the “Net cage” technique's potential for superior functional outcomes.

This study has limitations: it is a small-sample, single-center retrospective analysis without a control group, and only descriptive statistics were performed. The absence of modern patient-reported outcome measures (e.g., Kujala, Lysholm) and biomechanical/cadaveric validation also constrains conclusions. Future large-sample, multi-center randomized controlled studies are needed to confirm the technique's efficacy, alongside biomechanical research to validate its fixation strength.

## Conclusions

The “Net cage” technique is a safe and effective internal fixation method for inferior patellar pole fractures, with the advantages of straightforward surgical operation, reliable fixation stability, facilitation of early postoperative functional exercises, and favorable knee joint function recovery (satisfactory ROM and Bostman scores at follow-up). Given its ability to address the core challenge of stabilizing small, comminuted fragments, this technique shows promising clinical value. However, due to the limitations of the study design, further verification by large-sample, multi-center randomized controlled studies is required before its widespread clinical promotion.

## Data Availability

The original contributions presented in the study are included in the article/Supplementary Material, further inquiries can be directed to the corresponding author.
