# Supplementary material for: “Net cage” technique in the treatment of inferior pole patella fracture
Source: Front Surg. 2026 Mar 11;13:1648538. doi: 10.3389/fsurg.2026.1648538 (PMC13013521; doi:10.3389/fsurg.2026.1648538)
Supplement: Supplementary file 1 [file Datasheet1.pdf]

A 64-year-old male patient underwent surgical treatment for a left patellar fracture. A re-examination X-ray on the 2nd postoperative day showed that one bone pin had displaced, but the internal fixation had not completely failed, and the fracture reduction was acceptable. At the 9-month postoperative follow-up, the fracture reduction was satisfactory with successful union. This indicates that the two tension bands function synergistically—even if one tension band fails, the second one can still provide protective fixation for the fracture.

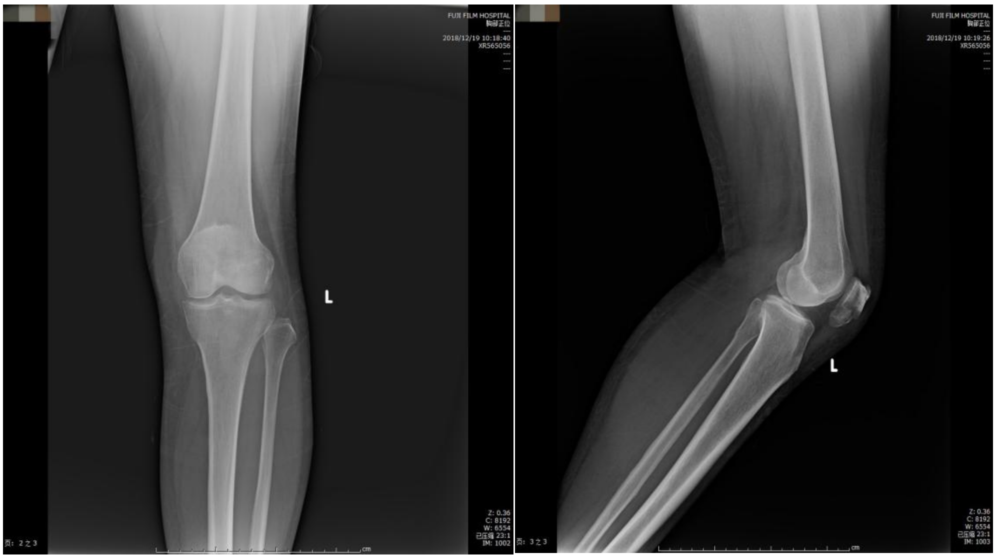

Preoperative anteroposterior and lateral X-ray films of the knee joint；

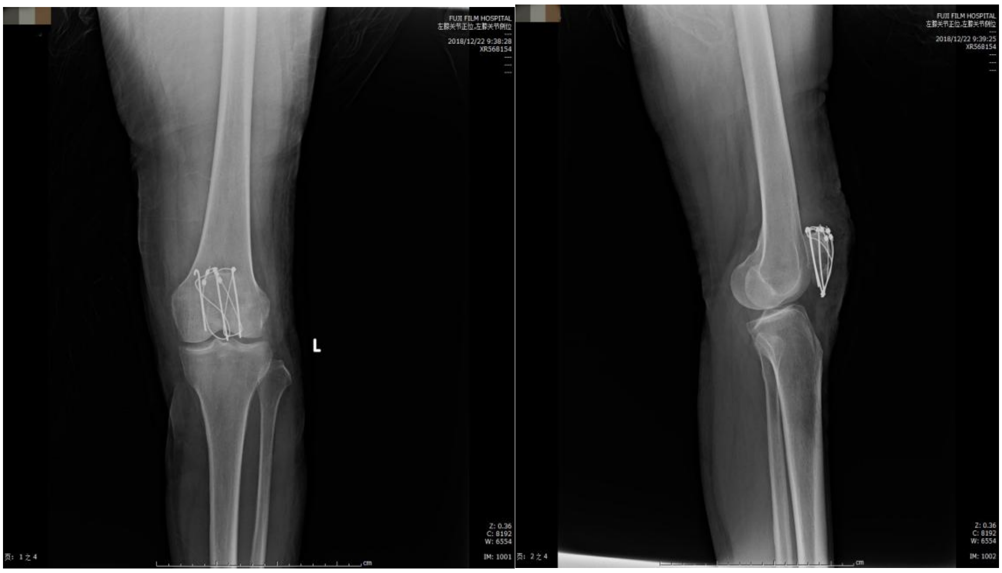

Anteroposterior and lateral X-ray films of the knee joint on the 2nd postoperative day；

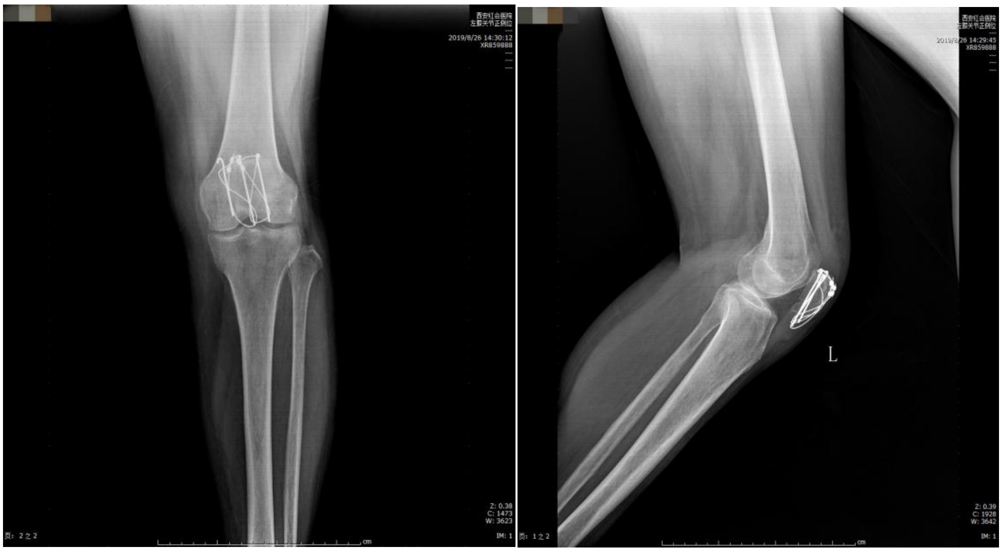

Anteroposterior and lateral X-ray films of the knee joint at the 9-month postoperative follow-up.

Table.1.a standardized rehabilitation protocol with quantified milestones.

Postoperative Time	Brace Angle	Active ROM Milestone	Quadriceps Activation	Weight-Bearing
Week 1-2	30° locked	≥30°	Isometric contraction (3 sets × 10 reps, 5s hold)	Partial (20-30% body weight)
Week 3-4	60° unlocked	≥60°	Isotonic contraction (3 sets × 12 reps, 0.5kg load)	Partial (50-60% body weight)
Week 5-6	Full range	≥90°	Eccentric training (3 sets × 10 reps, 1kg load)	Full (100% body weight)
Week 7-12	Discontinued	≥120°	Functional training (squats, step-ups)	Full + activity-specific
